# Straight aortic endograft in abdominal aortic disease

**DOI:** 10.1186/1749-8090-8-114

**Published:** 2013-04-29

**Authors:** Daniela Mazzaccaro, Maria Teresa Occhiuto, Giovanni Malacrida, Silvia Stegher, Andrea Raspadori, Stefano Manfrini, Domenico G Tealdi, Giovanni Nano

**Affiliations:** 1 First Unit of Vascular Surgery, IRCCS Policlinico San Donato, University of Milan, piazza E. Malan 1, San Donato Milanese, Milan 20097, Italy; 2 Department of Vascular Surgery, Casa di Cura Salus, Ferrara, Italy

**Keywords:** EVAR, Straight endograft, AAA

## Abstract

**Background:**

We describe our 8-year experience with the use of endovascular techniques (ET) for the treatment of abdominal aortic aneurysms (AAA) through a straight endograft.

**Methods:**

We retrospectively reviewed data of all patients who were treated for AAA using ET in two centres from 1998 to 2012 and who received a single straight endograft (group A) or a double straight tube (group B). Outcomes were analyzed to assess survival, absence of endoleak and absence of reintervention for both groups. Log-rank and Chi-Square were used as appropriate to make comparison between the two groups. P values < .05 were considered statistically significant.

**Results:**

Fifty-three patients from 1998 to May 2012 were treated for AAA using a straight endograft. In 28 cases (52.8%) a single aortic straight tube was used (Group A), while in the remaining cases a “double trombone technique” was used (Group B).

Primary success was obtained in 52 cases (98.1%). In one patient of group A immediately after the operation we observed a type Ia endoleak, which was correct with a proximal aortic cuff.

Fluoroscopy time, operation time, amount of intraprocedural contrast medium and blood loss were slightly higher for group B, even if not significantly. Mortality at 30 days was nil for both groups. Mean follow-up was 49 months (range 2–153 months).

Five patients died in group A, four of them for a neoplastic disease and the remaining for aortic rupture. No patients died in group B. Endoleaks occurred more frequently in patients of group A (5 type I endoleaks and 1 type II endoleak from a lumbar artery).

Reintervention were more frequent for patients of group A, being type I endoleak the main cause. A stent fracture was observed in a patient who received EVAR by “trombone technique” 3 months later. Reintervention was then necessary and a third stent was successfully placed to cover the lesion.

**Conclusions:**

In our experience the endovascular repair of AAA using straight aortic endografts was a safe and effective technique. Reintervention and endoleaks were slightly more frequent in patients who had received a single endograft compared to patients who were treated using the “trombone technique”.

## Background

Historically, the first endovascular repair of an abdominal aortic aneurysms (EVAR) in the western world was performed in 1990 by Dr. Parodi [1], who put a straight endograft into the abdominal aorta. Limitations of the use of a similar device soon became evident besides its clear advantages, as the straight tube lacked of both columnar support and distal sealing to ensure enough durability over time, causing migration and subsequent type I endoleak. To overcome this problem, a second balloon-expandable stent was introduced to give more support and distal sealing to the first tube [2].

However the marketing of new self-expandable materials and bifurcated endoprosthesis, which had moreover a smaller caliber than the previous ones, caused a halt to the spread of straight stents and balloon-expandable materials.

Indeed, these second generation grafts were not a panacea, as they showed a reduced reliability over time as well, resulting in high number of reintervention after EVAR in the medium and long term [3]. These are factors to be strongly considered during the decision to perform EVAR, as reinterventions represent adjunctive expenses for society.

In 2007, Ruppert et al. [4] reported their experience about a modified technique for deploying tubular straight grafts (“Trombone technique”), with results which compared favourably to those obtained using bifurcated grafts in terms of durability and freedom from endoleaks in the early and mid-term.

As in our institution straight aortic endografts are still used in well selected patients, we decided to critically review our 14-year experience in endovascular treatment of aortic aneurysms through the use of tubular endoprosthesis with both conventional and trombone technique.

## Methods

The research described in this manuscript was approved by the ethics committee at each respective institution and was conducted per the guidance set forth in the Declaration of Helsinki. Informed consent was obtained from each patient.

We retrospectively reviewed data of all patients who were treated for abdominal aortic aneurysm (AAA) using endovascular technique (ET) in our two centres from 1998 to May 2012 and who received a single tubular aorto-aortic endograft (group A) or a double straight tube (group B).

Single or double straight endograft were used in case of AAA which did not involve aortic bifurcation, with a good proximal neck (>15 mm long, presence of calcification or thrombus in less than 50% of vessel perimeter, <30 mm wide and with an angulation <60°) and a good distal neck (>20 mm long, with an angulation <55° and presence of calcification or thrombus <30 mm). Challenging anatomies were excluded.

**Figure 1:**
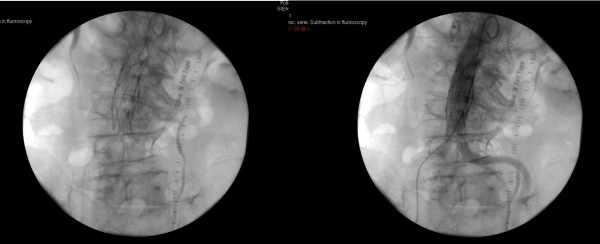
Placement of a single tube which completely excluded the AAA.

The single straight tube (Endologix®, Talent®, Vanguard®, Parodi® or Baxter Lifepath®) was placed according to the specific instructions for the device and to preoperative imaging data (Figure 1).

**Figure 2:**
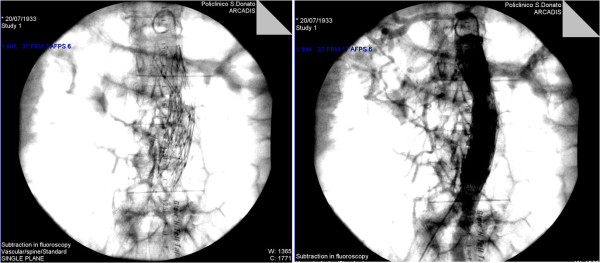
Trombone technique and complete exclusion of the AAA.

In group B, the two aortic tubes were placed using the “Trombone-technique” (TT, Figure 2): a first Endologix® aortic cuff is laid on the aortic carrefour. A second Endologix® aortic cuff with a proximal free-flow to enhance proximal fixation is deployed cranially to the first, next to the origin of the renal arteries. This second cuff, which has a slightly larger caliber, is imbricated with the previous one with 2.5-3 cm of overlap. A 20% oversizing is calculated for proximal and distal devices, while the length of the grafts is chosen so that the sum of their extent exceeds about 3 cm the distance between the origin of the distal renal artery and the aortic carrefour. The purpose of this technique is to ensure stability to the graft-system, which is integral with the aortic bifurcation.

All procedures were performed in the operating room using a mobile C arm. Endografts were deployed using mainly a surgical exposure of femoral vessels.

Long term follow-up was obtained from outpatient visits and computed tomography (CT) scan or duplex scans. Imaging was performed every three months during the first year after operation and therefore annually.

All patients who survived were also interviewed through telephone calls before statistical analysis, which was performed using the software JMP® 5.1.2 (SAS Institute Inc.).

Outcomes were analyzed to evaluate survival, presence of endoleak and reintervention for both groups. Primary success was defined as the absence of any type of perioperative endoleak.

Log-rank and Chi-Square tests were used as appropriate to make comparison between the two groups. P values < .05 were considered statistically significant.

## Results

Fifty-three patients from 1998 to May 2012 were treated for AAA using a straight endograft. Of them, 47 were men (88.6%). Patient’s mean age was 72.5 years (range 58–84).

Four patients had a neoplastic disease, as shown in Table 1.

**Table 1:** Patients’ characteristics and anatomical data of both groups

	**Single tube (n = 28)**	**Trombone Technique (n = 25)**	**P**
**Male Sex**	25 (89.3%)	22 (88%)	.13
**Mean age, years (Range)**	71.2 (67–84)	73.4 (58–82)	.42
**Aetiology**			
Atherosclerosis/Degeneration	21 (75%)	19 (76%)	.48
IMH	1 (3.6%)	1 (4%)	.16
PAU	3 (10.7%)	1 (4%)	.06
PSA	3 (10.7%)	4 (16%)	.07
**Comorbidities**			
Diabetes	13 (46.4%)	6 (24%)	.32
Coronary Artery Disease	9 (32.1%)	7 (28%)	.19
Previous stroke/TIA	0 (0%)	1 (4%)	.21
Hypertension	18 (64.3%)	10 (40%)	.12
Hypercholesterolemia	6 (21.4%)	4 (16%)	.45
Obesity	2 (7.1%)	1 (4%)	.21
COPD	5 (17.8%)	3 (12%)	.32
Neoplasm	4 (14.3%)	0 (0%)	.05
Current or previous smoke	18 (64.3%)	12 (48%)	.22
**Anatomical data (Mean ± SD), mm**			
Proximal aortic neck diameter	25 ± 1.8	24 ± 1.2	.15
Proximal aortic neck lenght	20 ± 5	21 ± 1.2	.21
Distal aortic neck diameter	22 ± 0.2	18 ± 0.7	.34
Distal aortic neck lenght	24 ± 1.2	10 ± 2	.07
Angulation of proximal aortic neck (degrees)	30° ± 7.5°	35° ± 12°	.09
Sac diameter (range)	5.2 (5.0-8.4)	5.1 (4.6-6.2)	.12
Aortic length	84 (60–120)	89 (75–114)	.08

* IMH = Intra Mural Hematoma.

PAU = Parietal Aortic Ulcer.

PSA = Pseudoaneurysm.

TIA = Transient Ischaemic Attack.

COPD = Chronic Obstructive Pulmonary Disease.

Forty AAA had atherosclerotic and degenerative aetiology, one of them was treated in an emergency setting due to fissuration. There were 4 penetrating abdominal aortic ulcers (one of them required emergent operation for hemodynamic instability in an obese man) and two intramural hematomas. The remaining were aortic pseudoaneurysms of proximal anastomosis in AAA which had previously been treated using open surgical repair (OSR).

Endologix® grafts were used mostly (40 cases, 75.4%), besides Vanguard® grafts (7 cases), Talent®, Parodi® and Baxter Lifepath® (2 cases each). In 28 cases (52.8%) a single aortic straight tube was used (Group A), while in the remaining cases a “double trombone technique” was used, as described previously (Group B).

Primary success was obtained in 52 cases (98.1%). In one patient of group A we observed a type Ia endoleak at the end of the procedure, which was immediately corrected: an aortic cuff was then placed proximally and the patient was discharged in 4^th^ post-operative day (POD).

Two patients of group A and two of group B respectively needed additional procedures during the operation. In particular, in both patients who received a single aortic tube, a short independent covered stent (Viabahn®) was placed to correct a left common iliac aneurysm. In group B, one patient underwent a right renal transluminal angioplasty for a severe stenosis, and a last patient needed a right common femoral endarterectomy previous to the aortic endografting.

Intraprocedural and in-hospital data are described in Table 2 for both groups. Fluoroscopy time, operation time, amount of intraprocedural contrast medium and blood loss were slightly higher for group B, even if not significantly. On the other side, in-hospital stay was similar for both groups. Neither blood transfusion nor intensive care unit was necessary in any case.

**Table 2:** Intraprocedural and in-hospital data (Mean ± SD)

	**Single tube (n = 28)**	**Trombone technique (n = 25)**	**P value**
**Time of operation (min)**	31.1 ± 3	42 ± 4	.06
**Amount of contrast (cc)**	26.5 ± 2.5	38.2 ± 3.4	.05
**Fluoroscopy time (min)**	9.2 ± 1.4	15.2 ± 2	.05
**Blood loss (cc)**	120 ± 13	152 ± 11	.06
**Adjunctive procedure**	2/28 (7.1%)	2/25 (8%)	.12
**Length of stay (days)**	3.2 ± 0.8	3.1 ± 0.5	.25

Mortality at 30 days was nil for both groups. A post-implantation syndrome occurred in 6 patients (4 of them had received a single aortic tube) during hospital stay and immediately resolved after administration of low-dose corticosteroids. We didn’t observe any other complication within 30-days (Table 3).

**Table 3:** Summary of early and late events during follow-up for both groups

	**Single tube**	**Trombone technique**	**P**
**30-days**			
**Endoleak I**	1/28 (3.6%)	0/25 (0%)	.09
**Post-implantation syndrome**	4/28 (14.3%)	2/25 (8%)	.22
**Reintervention**	1/28 (3.6%)	0/25 (0%)	.09
**Long term**			
**Endoleak I**	**5/28 (17.8%)**	**0/24 (0%)**	**.02**
**Endoleak II**	1/28 (3.6%)	0/24 (0%)	.08
**Graft infection**	1/28 (3.6%)	0/24 (0%)	.08
**Stent fracture**	0/28 (0%)	1/24 (4%)	.07
**Reintervention**	**5/28 (17.8%)**	**1/24 (4%)**	**.03**
**Rupture**	1/28 (3.6%)	0/24 (0%)	.08
**Death**	**5/28 (17.8%)**	**0/24 (0%)**	**.02**

Mean follow-up was 49 months (range 2–153 months).

Five patients died in group A, four of them for a neoplastic disease which was already present at the moment of EVAR. Death occurred in the 10^th^, 16^th^, 25^th^ and 31^st^ postoperative month respectively. The remaining patient died for aortic rupture after 28 months, but he had refused any visit after operation and we had follow-up data only through telephonic interview.

In group B no patients died and no aortic rupture occurred. Follow-up data were available for 24 out of 25 patients of this group, as one was lost.

Endoleaks occurred more frequently in patients who received a single straight tube (Table 3). In particular, we observed 4 type Ib endoleaks, 1 type Ia endoleak and 1 type II endoleak from a lumbar artery. Endoleaks were diagnosed respectively after 13, 25, 27, 60, 52 and 22 months from the operation. An adding cuff was placed in all cases of type I endoleaks, but complete exclusion of the aneurysmatic sac was achieved in 3 cases only. In the case of type Ia endoleak, the single tube had been placed to correct a proximal anastomotic pseudoaneurysm after OSR. Once the endoleak had been diagnosed, a second endovascular attempt was performed to exclude the leakage but symptoms of a serious graft infection became evident. Thus the patient underwent surgical explantation of all grafts after axillo-bifemoral bypass at 52 months.

In the remaining case, late conversion to open surgery was necessary for persistency of flow throughout the sac in the 60^th^ postoperative month. The patient with type II endoleak is still under semestral surveillance, as the aneurysmatic sac has not showed any sign of growth yet.

Reintervention was more frequent for patients of group A, being type I endoleak the main cause (Table 3). There were no reinterventions for graft thrombosis. A stent fracture was observed in a patient who received EVAR by “trombone technique” 3 months later. The patient was symptomatic for buttock and calf claudication. Reintervention was then necessary and a third stent was successfully placed to cover the lesion.

## Discussion

Many studies in the literature [5-7] demonstrated that endovascular treatment of abdominal aortic aneurysms (EVAR) was associated with lower postoperative mortality and morbidity rates compared to open surgery repair (OSR), but this advantage seemed to decrease at long term because of higher reintervention rates in EVAR group.

The overview of the patients who underwent EVAR, in fact, progressively worsened with the progression of follow-up: EVAR-1 [5] showed that after 4 years complication rate was five times higher than after OSR, being type I and type II endoleak, migration of the graft and graft thrombosis the main causes of complications. These factors lead mostly to reinterventions, which were three times more frequent. The same findings were reported by the DREAM [6] group during the first nine months of follow-up.

Wilt and Coll. too [7], in a large meta-analysis performed in 2006 showed that EVAR did not improve long-term survival or health status over OSR, though peri-operative outcomes were improved. Moreover EVAR was associated with more complications, need for reintervention, monitoring and costs compared to OSR or no intervention. Recent SVS Guidelines [8] substantially confirmed these findings.

Thus reintervention still remains the Achilles heel of EVAR, and this factor must be strongly considered as it represents adding cost for society, in a period in which the availability of increasingly expensive devices has to face the need for containment of the outgoings.

While acknowledging the inferiority of EVAR compared to OSR, Wilt and Coll. [7] admitted however that most of analyzed studies were performed using dating models of endoprosthesis and hoped future research about devices and techniques to reduce postoperative complications.

SVS guidelines too [8] advocated the need for further improvements in EVAR devices and related techniques to reduce long-term complications and overall cost.

The use of tubular devices, although previously consigned to history by the wide criticism about poor durability over time, has been recently recalled. Aorto-aortic tubes have a series of advantages over bifurcated and conical grafts. The implantation procedure usually takes less time and radiations and the overall cost is substantially reduced [9]. Tubular endografts are also less likely to occlude as they have no kinking or twisting, if compared to bifurcated and aorto-monoiliac devices [10]. In our series, in fact, we did not observe any graft thrombosis. This fact may lead to the reduction of reintervention due to graft occlusion, but concern remains about exclusion of the aneurysmatic sac over time. The single tube in fact may lack of both columnar support and distal sealing, causing mainly type Ib endoleak, as reported in our series. Moreover, type Ib endoleak can be due to aneurysmatic degeneration of the remaining portion of the pre-carrefour abdominal aorta. Implantation of a second stent-graft within the distal end of the first tube then has been proposed [11]. This modified technique (“trombone technique”) allowed the two stent-grafts to be implanted in the correct position relative both to the proximal and to the distal implantation site, thus minimizing the risk of type I endoleak.

In 2007 Ruppert et al. [4] reported their experience about the use of the trombone technique as a planned procedure for deploying tubular aorto-aortic stent-grafts. He described very promising early and midterm results, which compared favorably to those reported for bifurcated devices and open repair.

In 2008 Saratzis and Coll. [9] too evaluated this technique comparing outcomes of 45 patients electively treated for AAA using the trombone technique to 8 patients who received single tube grafts. They observed that the overall device-related complication rate was significantly lower for patients treated with the trombone technique (11% versus 75%, p = .001).

The results observed in our series are consistent with those reported by these authors.

In our series, in fact, we observed that all endoleaks and reinterventions at long term occurred in patients who were treated using a single straight endograft. On the other side, blood loss, fluoroscopy time and length of procedure were slightly lower if compared to those of “trombone technique”.

Making a comparison is difficult, however, given the small number of patients in our series, with a disease which is strictly limited to the infrarenal aortic segment. These factors are the major limitations of our study and do not allow to generalize the results obtained.

## Conclusions

In our experience the endovascular repair of AAA using straight aortic endografts was safe and effective. Reintervention and endoleaks were slightly more frequent in patients who had received a single endograft compared to patients who were treated using the “trombone technique”. On the other side, blood loss, intraoperative time of fluoroscopy and intervention time were slightly lower if a single tube had been used.

## Abbreviations

ET: Endovascular techniques; AAA: Abdominal aortic aneurysm; EVAR: EndoVascular aortic repair; CT: Computed tomography; OSR: Open surgical repair; POD: Post-operative day; SVS: Society of Vascular Surgery.

## Competing interests

The authors declare that they have no competing interests.

## Authors’ contributions

DM acquisition of data, analysis and interpretation of data, drafting of the manuscript. MTO acquisition of data, revision of the manuscript. GM acquisition of data, revision of the manuscript. SS acquisition of data, revision of the manuscript. AR conception and design of the study. SM conception and design of the study. DGT conception and design of the study. GN conception and design of the study, revision of the manuscript, final approval of the manuscript.
